# Charge-Competition AIEgens Induce Mitochondrial Dysfunction for Selective Eradication of *Candida albicans* while Restoring Vaginal Microbiota

**DOI:** 10.4014/jmb.2601.01074

**Published:** 2026-06-12

**Authors:** Runjie Zhang, Xiaoxue Li, Xinyi Chen, Aijun Shen, Xuemei Zhang, Chen Peng, Jin Qiu

**Affiliations:** 1 Department of Pharmacology, Fudan University, Shanghai, P. R. China; 2 Obstetrics and Gynecology Department, Tongren Hospital, Shanghai Jiao Tong University School of Medicine, Shanghai, P. R. China; 3 Department of Pathology, The Second Affiliated Hospital, Zhejiang University School of Medicine, Hangzhou, P. R. China; 4 Lingang Laboratory, Shanghai, P. R. China; 5 Department of Radiology, Shanghai Public Health Clinical Center, Fudan University, Shanghai, 201508, P. R. China

**Keywords:** TPE-ET, *Candida albicans*, Charge competition, Vaginal microecology, Vulvovaginal candidiasis

## Abstract

The principal therapeutic challenge in vulvovaginal candidiasis (VVC) is that the non-selective nature of conventional antifungal drugs, which frequently perturb vaginal microecological homeostasis and disrupt *Lactobacillus* barrier, lead to the emergence of recurrent infection and drug resistance. This study aims to develop novel antifungal agents capable of efficiently and selectively eradicating pathogenic fungi while protecting and promoting the growth of *Lactobacilli*, with real-time monitoring capabilities and significant potential for clinical application. Harnessing the principle of charge-competition, we engineered a cationic amphiphilic aggregation-induced emission luminogens (AIEgens, named as TPE-ET), where hydrophobic chain length served as a key determinant governing membrane affinity, aggregation propensity, and antimicrobial selectivity. This design empowered potent eradication of *Candida albicans* (*C. albicans*) while concomitantly favoring the proliferation of beneficial *Lactobacilli*. Moreover, TPE-ET disrupted *C. albicans* biofilms and suppressed virulence genes related to adhesion, invasion, and drug resistance. In a murine VVC model, TPE-ET reduced fungal burden by over 90%, facilitating the repair of damaged vaginal epithelium and the reconstitution of a *Lactobacillus*-dominat vaginal microbiome. Remarkably, TPE-ET outperformed clotrimazole in restoring healthy microecological balance, as manifested by diminished *Proteobacteria* abundance alongside increased *Firmicutes* (notably *Lactobacillus*) and *Bacteroidetes*. Mechanistic studies revealed that TPE-ET exerted its remarkable antifungal activity by targeting the mitochondrial inner membrane, disrupting the metabolism-inflammation axis and eliciting mitochondrial dysfunction. Collectively, this dual merits of membrane charge-selective targeting and AIEgens-mediated visualization established an innovative therapeutic strategy for VVC, featuring superior efficacy, exquisite selectivity, and real-time monitoring capability with significant clinical potential.

## Introduction

VVC represents one of the most prevalent mucosal fungal infections affecting women globally. This gynecological disorder arises from pathogenic yeast colonization with the vulvar and vaginal mucosa, with *C. albicans* identified as the predominant etiological pathogen. More than 70% of women worldwide experience at least one episode of VVC over their lifetime. Clinically, this condition is typified by hallmark manifestations including pruritus vulvae, abnormal vaginal discharge and concomitant injury to the vaginal mucosal epithelium [1, 2]. Worse still, 40% to 50% of cases have suffered from recurrence[3]. The symptoms and management of VVC substantially exert a substantial adverse impact on psychological and sexual health, significantly impairing quality of life. Despite the widespread application of antifungal therapies, issues of recurrence and drug resistance remain prominent. These limitations indicate that current strategies are inadequate in meeting clinical needs, highlighting an urgent demand for more selective and long-lasting treatment options.

Current VVC management relies primarily on azoles (*e.g.*, clotrimazole) and polyenes, which remain the first-line clinical choice via inhibiting ergosterol synthesis or binding to the fungal cell membrane to exert antifungal effects[4]. Nevertheless, these agents suffer from poor target selectivity. They not only eliminate *C. albicans* but also disrupt the delicate homeostasis of vaginal microbiota, inflicting damage on beneficial strains such as *Lactobacillus*. Furthermore, due to the structural similarity between fungal ergosterol and mammalian cholesterol, these drugs can adversely alter the membrane fluidity and permeability of normal epithelial cells to generate nonspecific toxicity[5, 6]. These combined actions disrupt the vaginal local microecological balance, impair the protective *Lactobacillus* barrier, diminsh the acidic microenvironment, and promote pathogen overgrowth. Given the crucial role of *Lactobacillus* in sustaining vaginal acidity, inhibiting pathogen colonization and regulating immune homeostasis, their loss directly compromises host defense, exacerbates inflammatory responses and significantly increases the risks of VVC recurrence and drug resistance [7, 8]. Consequently, achieving high efficiency and selective eradication of pathogenic fungi while concurrently protecting and promoting the growth of *Lactobacillus* is pivotal to overcome the current therapeutic challenges in VVC.

Fungal cell membranes possess a lower negative charge density in comparison with bacterial cell membranes. *C. albicans* displays a distinct difference in net surface charge in comparison with *Lactobacillus*, providing a theoretical foundation for achieving targeted antifungal activity [9, 10]. Membrane-targeting cationic amphiphiles are structurally composed of hydrophilic cationic groups and lipophilic segments. Their compounds preferentially bind to the fungal membrane through electrostatic attraction. Subsequently, their hydrophobic terminus inserts into and disrupt the membrane integrity, highlighting its promise as a potential antifungal candidates. Their cationic groups facilitate coulombic binding to negatively charged bacterial or fungal surfaces, whereas the lipophilic domains penetrate cell walls and membranes to exert microbicidal effects [11, 12]. Aggregated cationic amphiphiles possess elevated cationic charge density and localized concentration, promoting rapid electrostatic binding to anionic fungal envelopes and circumventing gradual accumulation [13]. The additional integration of AIEgens properties enables pathogen visualization via the light-up effect upon aggregation. This aggregated state further reinforces their membrane-disruptive efficacy and fungicidal activity. In this strategy, the design of the hydrophobic segment in the AIEgens is particularly critical. The length and configuration of hydrophobic chain directly govern membrane insertion depth and stability of aggregates. AIEgens with long hydrophobic chains, due to their larger molecular volume or higher aggregation density, may be retain at the surface of the biofilm or cell wall, resulting in reduced penetration capability and induces cytotoxicity [14, 15]. Their propensity for premature or overly tight aggregation can sterically hinder the contact between the cationic headgroups and the membrane, thereby compromising electrostatic binding efficiency. In contrast, shorter-chain AIE molecules can more readily penetrate the outer cell wall or biofilm, rapidly reach the lipid bilayer, and initiate membrane disruption. Their aggregation behavior is more moderate. This allows the cationic termini to interact more freely with the negatively charged fungal membrane, while the hydrophobic ends effectively embed into it, leading to a synergistic enhancement of membrane damage.

**Fig. 1:**
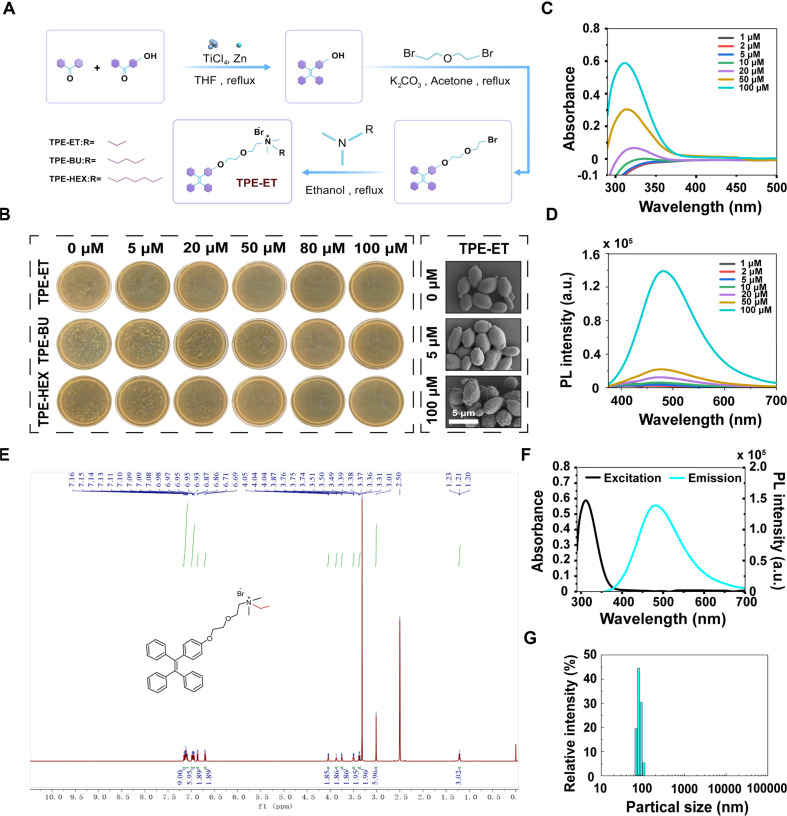
The physical properties of TPE-ET. (**A**) Schematic illustration of synthesizing TPE-ET. (**B**) A dose-dependent activity of three amphiphiles against *C. albicans* (left, the TPE-ET exhibited the strongest antifungal effect) and SEM morphological images of *C. albicans* incubated with different concentrations of TPE-ET (right). Scale bar: 5 μm. (**C**) The photoluminescence spectrum of TPE-ET ranged between 400 and 600 nm. (**D**) PL spectra of TPE-ET in DMSO/H_2_O mixture with different water fraction (fw), λex: 340 nm. (**E**) ^1^H NMR spectra of TPE-ET. (**F**) Ultraviolet absorption wavelength of TPE-ET in DMSO solution. (**G**) Size distribution of TPE-ET (10 μM) in H_2_O solution.

**Fig. 2:**
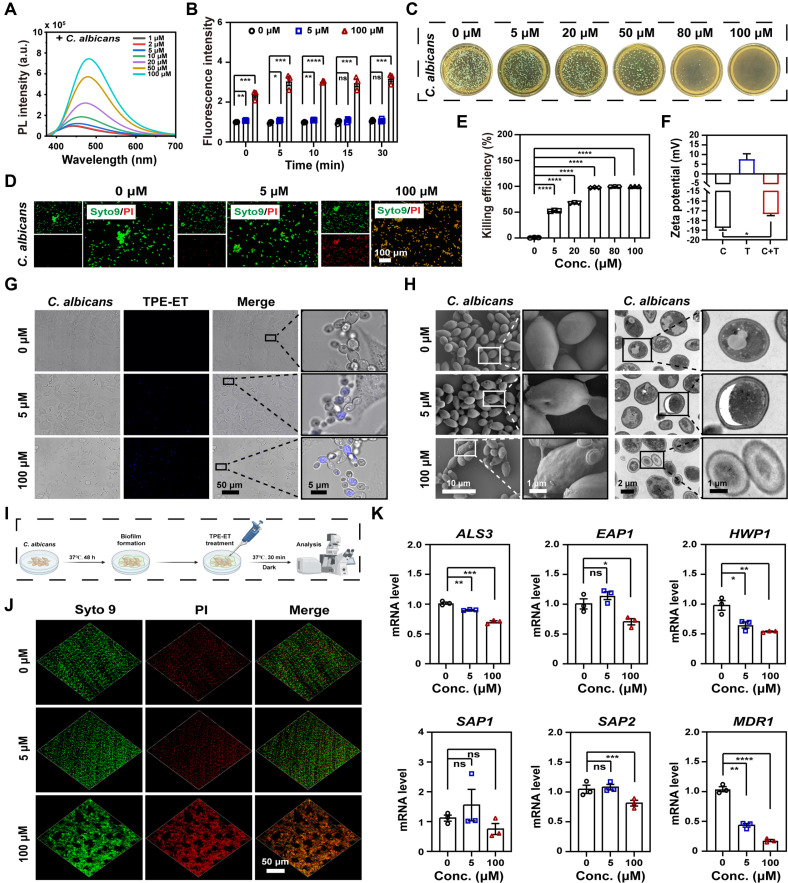
TPE-ET selectively killed *C. albicans* and inhibited its biofilm formation. (**A**) Photoluminescence spectra of mixtures of *C. albicans* and TPE-ET with different concentrations. (**B**) The fluorescence intensity of *C. albicans* incubated with TPE-ET at different concentrations was detected by fluorescence microplate reader in different time points (n = 3). 100 μM TPE-ET exhibited significant fluorescence intensity combined with *C. albicans*. (**C, E**) The antifungal activity of TPE-ET against *C. albicans* was positively correlated with its concentrations (n = 3). (**D**) Syto9/PI staining showed that the higher concentration of TPE-ET had a more previous killing effect on *C. albicans*. Scale bar: 100 μm. (**F**) The zeta potential results revealed that the binding of positively charged TPE-ET to the negatively charged *C. albicans* surface reduced the absolute value of the fungal zeta potential (n = 3). (**G**) Confocal fluorescence images of *C. albicans* specifically stained by TPE-ET for 30 min with VK2/E6E7 cell strain. Imaging conditions: 405 nm laser. Scale bar: 50 μm (left), 5 μm (right). (**H**) SEM and TEM morphological images revealed that the *C. albicans* incubated with higher concentration TPE-ET had more obvious signs of damage. Scale bar: 10 μm and 1 μm, respectively (left), 2 μm and 1 μm, respectively (right). (**I**) Scheme for detecting the biofilms formation ability of *C. albicans* upon TPE-ET treatment and the mRNA expression levels of biofilms-related genes. (**J**) Representative 3D images of *C. albicans* biofilms stained with Syto9/PI showed TPE-ET could disrupt biofilms formation. Scale bar: 50 μm. (**K**) The biofilms-related genes mRNA expression levels decreased in *C. albicans* treated with TPE-ET (n = 3). Statistical analyses were performed using two-way ANOVA (**B**), one-way ANOVA (E, K) and Student's t-tests (**F**). Error bars indicated the mean ± SD. Conc.: concentration. C: *C. albicans*. T: TPE-ET (50 μM). C+T: *C. albicans*+50 μM TPE-ET. ns: no significance, *p < 0.05, **p < 0.01, ***p < 0.001, and ****p < 0.0001.

**Scheme 1:**
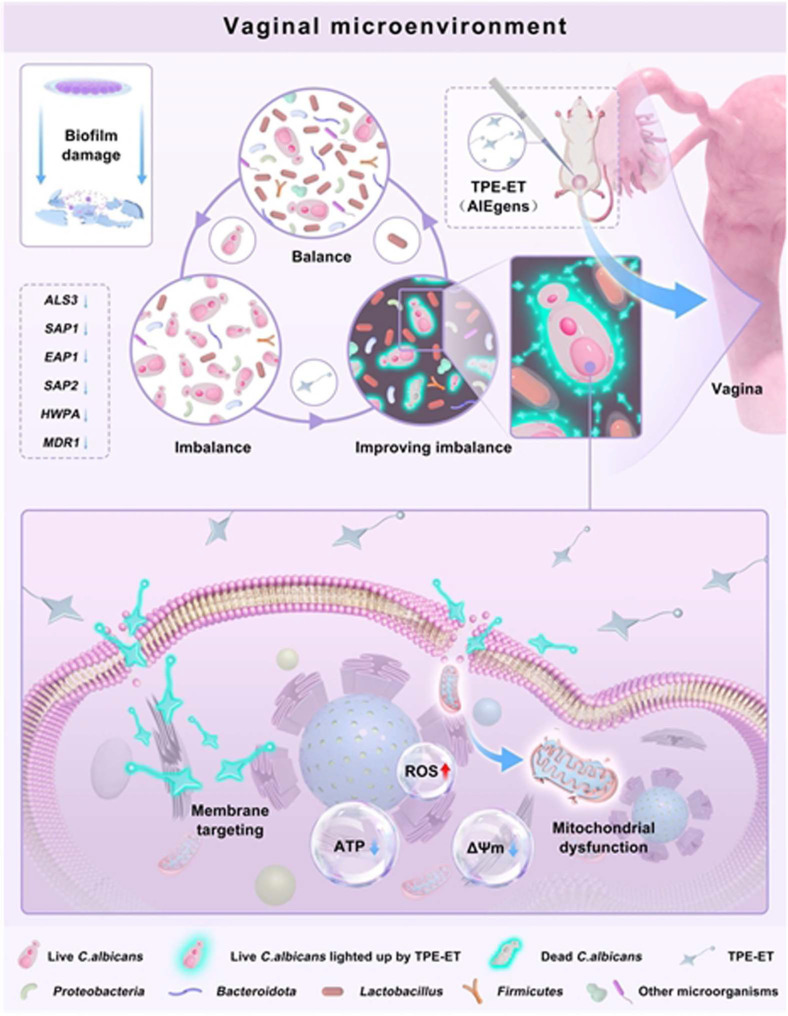
Schematic illustration for selective staining *C. albicans* and the relative killing mechanism by TPE-ET (TPE-ET targeted *C. albicans* based on membrane charge selectivity and AIE-mediated visualizability, cleared pathogenic fungi through inducing mitochondrial dysfunction and rehabilitated vaginal microbial homeostasis).

Based on above rationale, this study aims to develop a short hydrophobic-chain cationic amphiphilic AIEgens molecule based on the difference in surface membrane charge between bacteria and fungi, and name it TPE-ET with tetraphenylethylene (TPE) as the skeleton [16]. To achieve precise control over membrane binding and selective antifungal activity (Scheme 1), we rationally modulated the length of the hydrophobic alkyl chain. Results revealed that the TPE-ET (Fig. 1A) could rapidly light up and effectively kill *C. albicans* (Fig. 2) because of short hydrophobic chain [17, 18]. Meanwhile, TPE-ET promoted the growth of *Lactobacillus* and exhibited low toxicity towards vaginal epithelial cells. Furthermore, TPE-ET effectively disrupted *C. albicans* biofilms by reducing the transcriptional levels of genes involved in adhesion (*ALS3*/*EAP1*/*HWP1*), invasion (*SAP1*/*SAP2*), and drug resistance (*MDR1*) [19], reducing fungal drug resistance potential and maintaining the balance of vaginal internal environment. In a murine model of VVC, TPE-ET not only significantly reduced the fungal burden but also promoted vaginal mucosal repair, alleviated inflammation and restored the vaginal microecological balance.

## Materials and Methods

### Synthesis of TPE-ET, TPE-BU and TPE-HEX

(1) Synthesis of 4-(1,2,2-triphenylvinyl) phenol: A two-necked round-bottom flask was charged with benzophenone (1.82 g, 10 mmol), 4-hydroxybenzophenone (1.98 g, 10 mmol), and zinc dust (2.60 g, 40 mmol). After three cycles of evacuation and nitrogen backfilling, dry THF (70 mL) was added under N_2_. The mixture was cooled in a dry ice-acetone bath, and TiCl_4_ (2.2 mL, 20 mmol) was added dropwise with stirring. The reaction was then refluxed overnight under N2 and cooled to room temperature, 50 mL dilute hydrochloric acid (1 M) was added to the mixture, and then the mixture was extracted with dichloromethane (DCM). The combined organic phase was dried over anhydrous sodium sulfate and filtered. The crude product, obtained after solvent evaporation, was then purified via silica gel column chromatography with a gradient-free eluent system of n-hexane/ethyl acetate (40:1).

(2) To prepare (2-(4-(2-(2-bromoethoxy)ethoxy)phenyl)ethene-1,1,2-triyl) tribenzene, hydroxylated TPE (1.74 g, 5 mmol), dibromoethyl ether (1.39 g, 6 mmol), and K_2_CO_3_ (1.38 g, 10 mmol) were suspended in acetone (30 mL) and heated at reflux under nitrogen overnight. After the solvent was removed under reduced pressure, the crude residue was purified by flash column chromatography on silica gel, eluting with a 20:1 mixture of n-hexane and dichloromethane.

(3) Synthesis of TPE derivatives (TPE-ET, TPE-BU, TPE-HEX): A solution of the brominated precursor (2 mmol) and the respective amine (10 mmol) in anhydrous ethanol (20 mL) was refluxed under nitrogen atmosphere for 24 h. The reaction mixture was concentrated under reduced pressure, and the crude product was purified through sequential dissolution in minimal methanol followed by precipitation with excess tetrahydrofuran, yielding the target compounds as white powders after drying under vacuum.

### Sample Preparation and Characterization

Stock solutions (1.0 or 5.0 mM in DMSO) were prepared and subsequently diluted with deionized water to achieve the desired experimental concentrations. Structural confirmation was obtained by high-resolution mass spectrometry (HRMS, Finnigan MAT TSQ 7000, positive ion mode). Optical properties were characterized using UV-Vis spectroscopy (Milton Roy Spectronic 3000 array spectrophotometer) and fluorescence spectroscopy (PerkinElmer LS 55 luminescence spectrometer).

### *Lactobacillus* and *C. albicans* Culture

*Lactobacillus* (ATCC33820) and *C. albicans* (ATCC10231) were respectively inoculated in MRS medium agar plates and *C. albicans* selective medium agar plates, a single colony was obtained via an inoculation loop. Afterwards, a single colony was inoculated into fresh MRS broth and separately into SDB broth, then both were cultured overnight for subsequent use. *C. albicans* was cultured under microaerophilic conditions.

### Live and Dead Staining of *C. albicans* and *Lactobacillus*

*C. albicans* and *Lactobacillus* were incubated with propidium iodide (PI) and Syto9 for 30?min after treatment with TPE-ET. The cells received three rinses with PBS and examined under a confocal fluorescence microscope. Syto9 was excited at 480?nm and detected at 500 nm, while PI was excited at 490 nm and detected at 635 nm.

### Anti-*C. albicans* and *Lactobacillus* Activities Tests

The spread plate method was used to assess TPE-ET activity against *C. albicans* and *Lactobacillus*. Microbial suspensions were treated with TPE-ET (0, 5, 20, 50, 80, 100 μM) for 30 min at room temperature, then diluted (1:1500 for *C. albicans*, 1:800 for *Lactobacillus*). Aliquots (100 μL) were spread on CCM or MRS agar and incubated (24 h; 30°C for *C. albicans*, 37°C for *Lactobacillus*). Colony counts were recorded, and killing efficiency (%) = [(Control mean - Treated mean)/Control mean] × 100.

### Fluorescence Intensity Detection

*C. albicans* and *Lactobacillus* were incubated with different TPE-ET concentrations (0, 5, 100 μM) in different time points (0, 5, 10, 15, 30 min), then measured using the Varioskan LUX modular multimode reader Excitation (wavelength: 480 nm).

### Recognition of *C. albicans* and *Lactobacillus* after TPE-ET Treatment

Recognition of *C. albicans* and *Lactobacillus* treated with TPE-ET were conducted by confocal fluorescence microscope. *C. albicans* and *Lactobacillus* suspension were incubated with different TPE-ET concentrations (0, 5, 100 μM) for 15 min at room temperature. Images were taken by confocal laser microscopy (TCS SP8 SR, Leica, Germany) (λex = 405 nm).

### Biofilm Damage Examination

*C. albicans* in the logarithmic growth phase were seeded onto cell-adherent slides and incubated at 37°C for 48-72 h to form biofilms. The cells received three rinses with PBS, mature biofilms were exposed to 0, 5, or 100 μM TPE-ET for 30 min. The biofilms were then stained with Syto 9 and PI for 30 min respectively, followed by washing PBS with three times. Confocal laser scanning microscopy (CLSM) was used to capture biofilm images, which were then analyzed using NIS-Elements AR software. Syto 9 and PI were detected at Ex/Em of 480/490 nm and 500/635 nm, respectively.

### Co-Culture System of Cells and *C. albicans*

VK2/E6E7 cells and *C. albicans* were co-cultured with TPE-ET to evaluate the selective recognition ability of *C. albicans* rather than cells. VK2/E6E7 cells were cultured in confocal dishes at 1 × 10^5^ cells per dish overnight. *C. albicans* were incubated with TPE-ET at different concentrations (0, 5, 100 μM), which were added into the dish in order. After incubation for 15 min, images were taken under confocal laser microscopy (TCS SP8 SR, Leica, Germany) (λex = 405 nm).

### SEM Detection

*C. albicans* suspensions were exposed to TPE-ET (5 or 100 μM) for 30 min, washed thrice with PBS and fixed in 4% paraformaldehyde overnight. Dehydration of the samples was performed using a graded ethanol series, transitioned to isoamyl acetate and critical-point dried. After being placed onto scanning electron microscope (SEM) stubs, the dried samples were coated with gold via sputtering for 30 sec and then observed under the Hitachi SU8100 field-emission scanning electron microscope (Japan).

### TEM Detection

*C. albicans* suspensions were treated with TPE-ET (5 or 100 μM, 30 min), washed thrice with PBS, and fixed in 4% paraformaldehyde. Fixed specimens were embedded in 1% agarose, dehydrated through an ethanol gradient (30-100% in 7 steps, 15 min each), and transferred to embedding molds. After overnight curing at 37°C, blocks were polymerized at 60°C (over 48 h). Ultrathin sections (60-80 nm) were cut using an ultramicrotome, mounted on 150-mesh formvar-coated copper grids, stained, and imaged with a Hitachi HT7800 transmission electron microscope.

### Cell Cytotoxicity Examination

The cytotoxic potential of TPE-ET was examined in VK2/E6E7 cells plated at 1×10^4^ cells/well. After 30 min treatment with gradient concentrations (0-100 μM) and 24 h incubation, viability was quantified via CCK-8 assay (OD_450_).

### Zeta Potential Measurements

*C. albicans* were pelleted by centrifugation (3,000 ×g, 5 min), washed with PBS and resuspended. Cell suspensions were treated with TPE-ET (0-50 μM) for 30 min before zeta potential analysis using the Malvern Zetasizer.

### Flow Cytometry Detection of *C. albicans* Intracellular ROS and Fluorescence Change

Intracellular ROS levels in *C. albicans* and *Lactobacillus* were quantified by flow cytometry. Cell suspensions were stained with 10 μM DCFH-DA (1:1000 dilution) at 37°C for 30 min, followed by treatment with TPE-ET (5 or 100 μM, 30 min). Fluorescence intensity was measured using a BD FACS Vantage SE flow cytometer (BD Biosciences, USA).

### RNA-seq

Following 30 min exposure of *C. albicans* to TPE-ET, total RNA was isolated using RNA-easy Isolation Reagent (Vazyme, R701-01), and transcriptome sequencing was conducted by OE Biotech (China). Genes with significant differential expression were identified based on |log_2_FC| > 1 and *p* < 0.05, and the results were displayed using a volcano plot. Hierarchical clustering of DEGs was displayed in a heatmap comparing control and treated groups. Pathway enrichment analysis was conducted using GSEA with MSigDB gene sets (GO/KEGG).

### Intracellular ATP Level Measurement

The intracellular ATP concentration of fungus was ascertained using a commercially available ATP determination kit (Servicebio, G4309-96T). Fungus was lysed in extraction buffer, homogenized (5 min), and heat-denatured (2 min, 100°C). After centrifugation (10,000 ×g, 10 min, 4°C), supernatants were collected and immediately placed on ice. ATP content was determined by chemiluminescence measurement using a microplate reader.

### Mitochondrial Membrane Potential Detection

Mitochondrial membrane potential was measured by JC-1 staining (MCE, HY-K0601). The *Lactobacillus* and *C. albicans* treated with 100 μM TPE-ET were suspended in 500 μL PBS supplemented with 500 μL of 1 μM JC-1 solution for 30 min at 37°C in the dark. Then, *Lactobacillus* and *C. albicans* were centrifuged and washed with PBS three times, resuspended in PBS, followed with flow cytometry analysis at green fluorescence: Ex/Em = 490/525 nm, red fluorescence: Ex/Em=525/590 nm. The data were analyzed using FlowJo VX software.

### Establishment of Murine VVC Model

VVC models were established in 6-week-old female BALB/c mice with approval from Tongren Hospital Ethics Committee, Shanghai Jiao Tong University School of Medicine (A2025-005-01). Mice were randomized into groups (n = 3/group) and received subcutaneous β-estradiol to induce pseudoestrus. Vaginal inoculation was performed with 20 μL *C. albicans* suspension (2 × 10^9^ CFU/mL), followed by 15 min trendelenburg positioning. Mucosal healing was documented photographically on day 0, 1, 3, 5, and 7. Vaginal lavage fluid was collected and serially diluted (1:10) for analysis.

### Histological Experiments

Following a 7-day treatment period, vaginal tissues were collected from euthanized mice. The whole vaginal tract was dissected, post-fixed in 10% neutral buffered formalin, dehydrated in a graded ethanol series, and paraffin-embedded. Thin sections (4 μm) were cut and stained with H&E and PAS. For immunostaining, sections were incubated with primary antibodies (anti-IL-4, anti-IL-6, anti-IL-1β; Servicebio, China) overnight at 4°C, followed by secondary antibodies. Fluorescence images were captured using a Leica SP8 confocal microscope, and quantified with ImageJ (NIH).

### ELISA Measurement of Inflammatory Cytokines

Vaginal lavage fluid was collected via sterile pipettes, suspended in 100 μL sterile PBS (pH 7.2), and centrifuged (3,000 ×g, 10 min, 4°C). The supernatant was aliquoted and stored at -80°C. Cytokine levels (*IL-4*, *IL-6*, *IL-1β*) in samples from distinct treatment groups were quantified using ELISA kits (Servicebio) per manufacturer's protocol.

### ITS and 16S Sequencing

Fungal genomic DNA was extracted using the DNeasy PowerSoil Kit (Qiagen). Sequence reads were taxonomically classified against the SILVA database (v138) via q2-feature-classifier (default parameters). Amplicon sequencing and bioinformatics analysis were performed by OE Biotech. Alpha diversity metrics (Chao1 richness, Shannon diversity) were compared between control and TPE-ET treatment groups.

### Statistical Analysis

All values were shown as mean ± standard deviation (SD). Differences between two groups were analyzed with unpaired two-tailed Student's t-tests. To compare more than two groups, one-way and two-way ANOVA were adopted. The threshold for significance was *p < 0.05, **p < 0.01, ***p < 0.001, and ****p < 0.0001.

## Results

### Molecular Engineering, Fabrication and Characterization

Destroying pathogen membranes and cell walls is a potent antimicrobial strategy. Cationic amphiphile aggregates outperform monomers by first electrostatically binding to fungal surfaces, then releasing monomers to penetrate membranes [16]. Optimizing side-chain length enhances membrane permeability [20, 21], while alkyl chain engineering precisely modulates hydrophobicity [22]. To enhance water solubility and flexibility, alkoxy chains of varying lengths (ethane, butane, and hexane) were incorporated, yielding the corresponding derivatives TPE-ET, TPE-BU and TPE-HEX, respectively (Fig. 1A). The antifungal activity of TPE-ET, TPE-BU and TPE-HEX was estimated by targeting *C. albicans*. Dose-responsive antifungal effects of amphiphilic compounds on *C. albicans* were assessed using plate colony counting. The killing efficiency towards *C. albicans* was rapidly boosted with the increasing concentration of three amphiphiles. TPE-ET exhibited rapid, concentration-dependent killing efficiency against fungi, increasing from 52.63% at 5 μM to 97.60% at 50 μM, and achieving near-complete eradication (99.87%) at 100 μM (Fig. S1). Among them, TPE-ET exerted the highest antifungal activity towards *C. albicans*. For the untreated control group, the intact and smooth *C. albicans* were observed. In contrast, after treated with TPE-ET, the structures of *C. albicans* are collapsed and merged, and the fungal membranes are obviously disintegrated (Fig. 1B). Typical AIE properties were observed in TPE-ET upon incorporation of the TPE moiety and emited weakly below 50 μM, but a strong emission peak at about 476 nm appeared with the increase of concentration up to 100 μM, exhibiting distinct AIE behavior resulting from sub-micrometer aggregate formation (Fig. 1C). In subsequent phases, TPE-ET was selected for further antifungal investigation. Characterization revealed that one main absorption peak of ultraviolet absorption wavelength at about 340 nm was observed for TPE-ET in DMSO solution. The photoluminescence spectrum of TPE-ET ranged between 400 and 600 nm (Fig. 1D and 1F). Structural elucidation of TPE-ET was achieved using ¹H NMR spectroscopy coupled with high-resolution mass spectrometry (HRMS) (Fig. 1E, Fig. S2). Dynamic light scattering demonstrated TPE-ET forms around 100 nm aggregates with a narrow size distribution in water (Fig. 1G).

### Killing of *C. albicans* with TPE-ET

AIEgens exhibits weak or negligible emission in molecular dissolved states but become highly emissive upon aggregation [23]. Upon mixing different concentrations of TPE-ET with *C. albicans* and incubating for 30 min, the emission peak of TPE-ET gradually intensified. As the concentration increased to 100 μM, a strong emission peak emerged at approximately 476 nm (Fig. 2A). It was observed that TPE-ET penetrated into the *C. albicans* and showed obviously enhanced fluorescence emission in Fig. 2B, which demonstrated characteristic AIE upon aggregate formation. To optimize the vitro antifungal activity of TPE-ET, *C. albicans* were treated with different TPE-ET concentrations in dark condition for 30 min using the plate colony-counting method. After photographing the plates, the killing efficiency was computed.b As shown in Fig. 2C, *C. albicans* can be completely killed at 100 μM TPE-ET. Killing efficiency statistics were shown in Fig. 2E. *C. albicans* were counterstained with SYTOX Green/PI after 30 min incubation with TPE-ET[24], TPE-ET enhanced PI uptake in *C. albicans* while reducing Syto9 fluorescence in a concentration-dependent manner (Fig. 2D). Furthermore, zeta potential measurements revealed a distinct positive shift in the surface charge of *C. albicans* upon addition of 50 μM TPE-ET, with values increasing from -18.77±0.2333 mV to -17.35±0.1500 mV, potentially explained by the partial exposure of the ammonium head of TPE-ET molecules on the *C. albicans* surface (Figs. 2F, S3), suggesting that it inserted into the fungal membrane or entered fungus [25]. To determine whether TPE-ET exhibited selective lighting-up behavior, fluorescence imaging was utilized to monitor the interaction of TPE-ET concentration at 5 μM and 100 μM with the co-culture system of VK2/E6E7 and *C. albicans*. As shown in Fig. 2G, TPE-ET achieved efficient labeling of *C. albicans* and yielded strong fluorescence emission. But fluorescence signal was not observed in normal vaginal epithelial cell VK2/E6E7, suggesting the selective light-up behavior of TPE-ET. Electron microscopy analysis further revealed compromised morphological integrity in TPE-ET-treated *C. albicans*, with SEM imaging showing characteristic cell shrinkage and surface fissures (Fig. 2H left), subcellular structure changes of *C. albicans* treated with TPE-ET were observed by transmission electron microscopy (TEM)(Fig. 2H right). Control group fungus exhibited characteristic oval morphology with densely packed, homogeneous cytoplasm and intact organelles. Treated with 5 μM TPE-ET resulted in discontinuous cell walls and uneven cytoplasm. 100 μM TPE-ET caused cytoplasmic content reduction accompanied by peroxisomal accumulation of electron-opaque inclusions in *C. albicans*. These findings indicated that TPE-ET compromised fungal cell wall and membrane integrity, resulting in cytoplasmic leakage. Thus, TPE-ET exerted the antifungal activity to disrupt fungus membranes via intercalation and rapid fungal internalization that enables efficient killing of *C. albicans*.

### Fungal Biofilm and Drug Resistance

We examined the effect of TPE-ET on the clearance of *C. albicans* biofilms (Fig. 2I). As shown in Fig. 2J, the strong red fluorescence signal of *C. albicans* was detected after treated with 100 μM TPE-ET, indicating TPE-ET could permeate fungal biofilms and eradicate biofilm-embedded fungi. We also quantified expression of biofilm-associated genes in *C. albicans* using RT-QPCR (Primer sequences were provided in Supplementary Table S1). As shown in Fig. 2K, TPE-ET treatment resulted in dose-dependent suppression of all examined target gene expression. *ALS3*, *EAP1* and *HWP1* are fungal adhesion that structurally mimics host cadherins, critically orchestrating the hyphal transition and biofilm maturation in *C. albicans*[26]. *MDR1* encodes Mdr1p, the predominant efflux transporter in *Candida* species' major facilitator superfamily (MFS) and a key clinical resistance determinant in *C. albicans* [27]. Secreted aspartic proteases *SAP1* and *SAP2* promote invasive penetration by releasing hydrolases onto host cell surfaces during biofilm development, relating to the invasiveness of *C. albicans* [28]. The above results showed that TPE-ET could eliminate the biofilm of *C. albicans*, simultaneously downregulated the expression of genes related to adhesion, invasion and drug resistance.

### Effects of TPE-ET on *Lactobacillus*

**Fig. 3:**
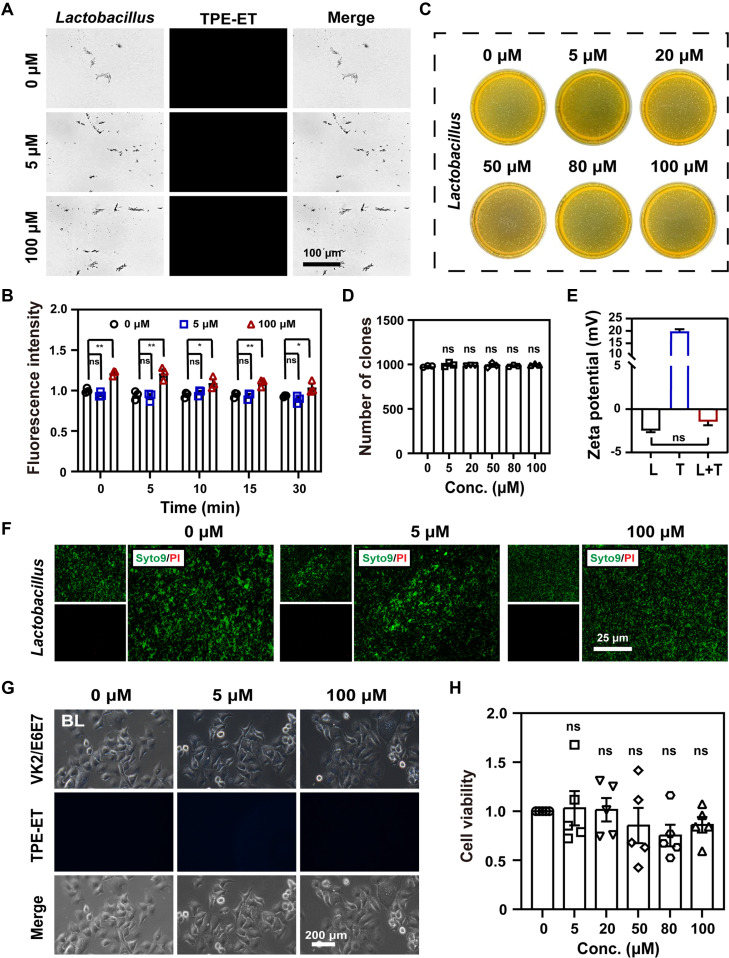
TPE-ET exerted no obvious effect on *Lactobacillus*. (**A**) Confocal fluorescence images of *Lactobacillus* stained by TPE-ET (5 μM and 100 μM, respectively) for 30 min were observed no fluorescence signal. Imaging conditions: 405 nm laser. Scale bar: 100 μm. (**B**) The fluorescence intensity of *Lactobacillus* incubated with TPE-ET at different concentrations detected by fluorescence microplate reader in different time points (n = 3). (**C and D**) TPE-ET at varing concentrations failed to affect the growth of *Lactobacillus* (n = 3). (**E**) Zeta potential results demonstrated the zeta potential of *Lactobacillus* remained unchanged following co-incubation with TPE-ET due to that TPE-ET failed to bing to *Lactobacillus* (n = 3). (**F**) Syto9/PI staining results showed that TPE-ET treatment caused no lethal damage to *Lactobacillus*. Scale bar: 25 μm. (**G**) Microscopic images showed no fluorescent signal in VK2/E6E7 cells after TPE-ET treatment, indicating that there was no interaction between TPE-ET and VK2/E6E7 cells. Scale bar: 200 μm. (**H**) CCK8 assays showed that no damage in VK2/E6E7 cells treated with different concentrations of TPE-ET for 24 h (n = 5). Statistical analyses were performed using two-way ANOVA (**B**), one-way ANOVA (D, H) and Student's t-tests (**E**). Error bars indicated the mean ± SD. Conc.: concentration. L: *Lactobacillus*. T: TPE-ET (100 μM). C+T: *Lactobacillus*+100 μM TPE-ET. ns: no significance, *p < 0.05, **p < 0.01.

To verify the antifungal properties of TPE for subsequent antifungal research, we studied whether TPE-ET affected the activity of dominant vaginal flora *Lactobacillus*. Vaginal *Lactobacilli* prevents dysbiosis by maintaining ecological equilibrium and blocking microbial encroachment [8]. The fluorescence imaging of *Lactobacillus* incubated with TPE-ET were shown in Fig. 3A, TPE-ET could not stain *Lactobacillus* with any emission. Fluorescence measurements results found a significant increase in fluorescence intensity at 100 μM concentration of TPE-ET. The increased fluorescence might ascribe to the enhanced aggregation of TPE-ET, which was consistent with the PL data (Fig. 3B). We used the plate colony-counting method and Syto9/PI staining to verify whether TPE-ET killed *Lactobacillus* [29, 30]. As shown in Fig. 3C and 3D, *Lactobacillus* colonies did not change significantly regardless of the culture time treated with concentration ranged from 5-100 μM. It has been reported that there are differences in surface membrane potentials among bacteria, fungi, and mammals. As shown in Fig. 3E and Fig. S4, zeta potentials of *Lactobacillus* incubated with different concentrations of TPE-ET were measured (10, 20, 50 and 100 μM). The zeta potential value of pure *Lactobacillus* was -2.487±0.1713 mV, while the values were -1.444±0.4118 mV after incubated with 100 μM TPE-ET. Zeta potential analysis demonstrated no obvious surface potential change of *Lactobacillus* caused by TPE-ET (100 μM). Given the differential surface charge properties, TPE-ET could selectively distinguished *C. albicans*. As well as in Fig. 3F, Syto9 could stain live *Lactobacillus* with green emission and PI staining displayed only weak fluorescence signal, indicating TPE-ET hardly affected the viability of *Lactobacillus*. This feature played a key role in enabling subsequent selective fungal killing with minimal side effects in antifungal research. This property was great significance for the purpose of selectively killing fungi and maintaining the balance of beneficial bacteria in the vagina in subsequent antifungal research. Moreover, TPE-ET did not obviously change the cell morphology and density of VK2/E6E7 cells (Fig. 3G). In addition, the CCK8 results showed cell viabilities of VK2/E6E7 cells had no significant decrease after incubation with TPE-ET at concentrations below 100 μM for 24 h (Fig. 3H), implying the potential of TPE-ET as a promising anti fungi substance *in vitro*.

### *In Vivo* Efficacy of TPE-ET against VVC

**Fig. 4:**
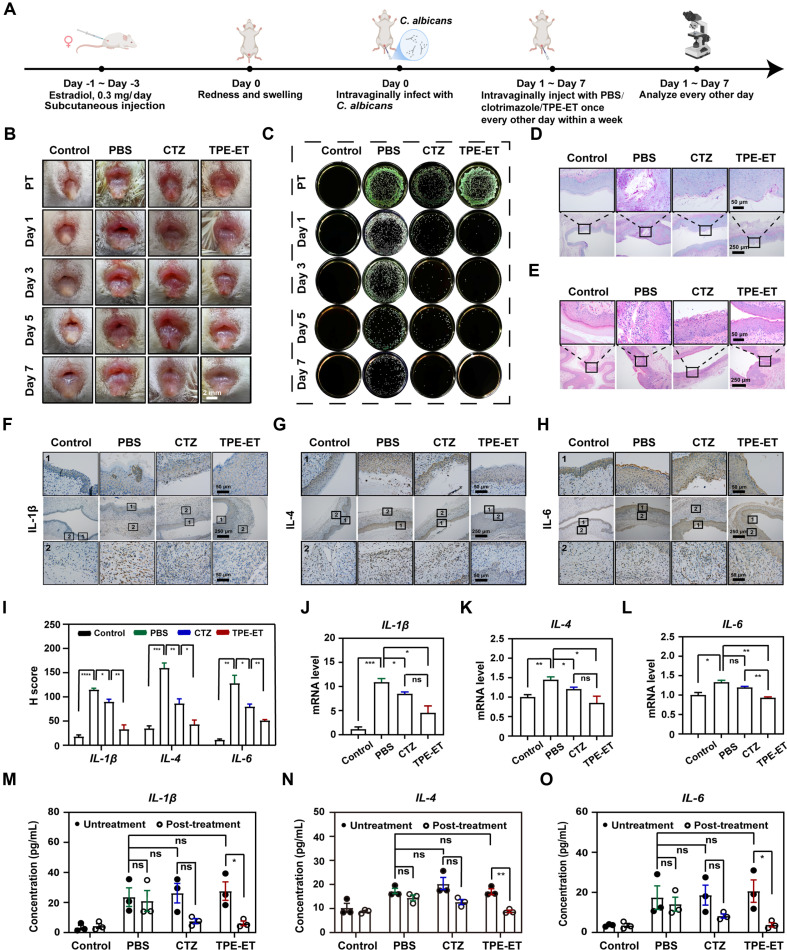
Antifungal effects of TPE-ET on VVC *in vivo*. (**A**) Schematic illustration of murine VVC model establishment, treatment (PBS/CTZ/TPE-ET) and detection procedures. (**B**) Representative images of murine vaginas from different treatment groups (control, PBS, CTZ and TPE-ET) within 7 days. Scale bar: 2 mm. (**C**) Agar images of vaginal lavage fluid from different treatment groups within 7 days. TPE-ET remarkably suppressed the proliferation of *C. albicans* in VVC mice. (**D**) PAS-staining images and (**E**) H&E-staining images of the vaginal tissue in different treatment groups. The amount of *C. albicans* on the surface of vaginal squamous epithelium was markedly reduced, accompanied by alleviated inflammation in the TPE-ET group. Scale bar: 50 μm (up), 250 μm (down). (**F to I**) The levels of the proinflammatory cytokines (**F**) *IL-1β*, (**G**) *IL-4*, (**H**) *IL-6* were detected by immunohistochemistry and H score(**I**) (n = 3). Scale bar: 50 μm (up and down), 250 μm (middle). QPCR (**J to K**) and ELISA (**M to O**) detection of *IL-1β*, *IL-4* and *IL-6* expression (n = 3). Statistical analyses were performed using two-way ANOVA (**I, M-O**) and one-way ANOVA (**J-L**). Error bars indicated the mean ± SD. CTZ: clotrimazole. ns: no significance, *p < 0.05, **p < 0.01, ***p < 0.001, and ****p < 0.0001.

Given its promising therapeutic potential against VVC *in vitro*, we further evaluated the antifungal efficacy of TPE-ET *in vivo*. *C. albicans*-induced murine VVC models were established, with uninfected healthy mice serving as controls. The clotrimazole (CTZ) group served as a clinically relevant comparison. The experimental workflow was outlined in Fig. 4A. Female BALB/c mice received daily subcutaneous injections of estradiol benzoate for the first three days. Vaginal washes for vaginal smears with majority of them was oval nucleated epithelial cells in proestrus, while in estrus was flaky, with keratinized epithelial cells accounting for the majority (Fig. S5). *C. albicans* suspensions were then intravaginally inoculated to establish the VVC model. Gram staining results showed mycelium and spores in vaginal smears, indicating successful establishment of the model (Fig. S6). Subsequently, PBS, CTZ, or TPE-ET were injected intravaginally every other day for 7 days. Fig. 4B and 4C demonstrated that *C. albicans*-infected mice exhibited vaginal erythema, edema with discharge, and abundant fungal colonization as evidenced by confluent growth on agar plates. Vaginal healing was observed in CTZ-treated mice by day 5 post-treatment, whereas TPE-ET administration resulted in significantly attenuated inflammation, with near-complete resolution of erythema, edema, and pathological discharge, the cell viability and colony formation of *C. albicans* treatments demonstrated a marked decline in vaginal washes on day 1. The mucosal defect healed on day 3. H&E-stained sections revealed no discernible morphological alterations in major organ following TPE-ET administration (Fig. S7). Periodic acid-Schiff (PAS) staining was conducted to evaluate fungal residue on the infected tissues of the mice, the number of mycelium and spore residues treated with CTZ were significantly reduced than that in the PBS group, and the TPE-ET treatment group had the least residual (Fig. 4D). H&E-stained sections exhibited significant disruption of mucosal architecture in PBS-treated mice, accompanied by robust neutrophil infiltration within the submucosal stroma. However, TPE-ET treatment group had less infiltration of vaginal inflammatory cells (Fig. 4E). *C. albicans* vaginal infection triggered a robust inflammatory cascade, characterized by significantly upregulated proinflammatory mediators *IL-4*, *IL-1β* and *IL-6* [31]. Immunohistochemistry (Fig. 4F-4I) and RT-QPCR results showed the expression level of *IL-4*, *IL-1β* and *IL-6* significantly decreased in TPE-ET treatment group (Fig. 4J-4L, Primer sequences in Supplementary Table S1). In addition, Elisa analysis showed TPE-ET significantly reduced *IL-4*, *IL-1β* and *IL-6* levels in vaginal washes compared with mice treated with CTZ (Fig. 4M-4O). These results indicated that the excellent antifungal effects of TPE-ET in killing *C. albicans* strains.

### Vaginal Microbiome Amelioration after TPE-ET Treatment Versus CTZ

**Fig. 5:**
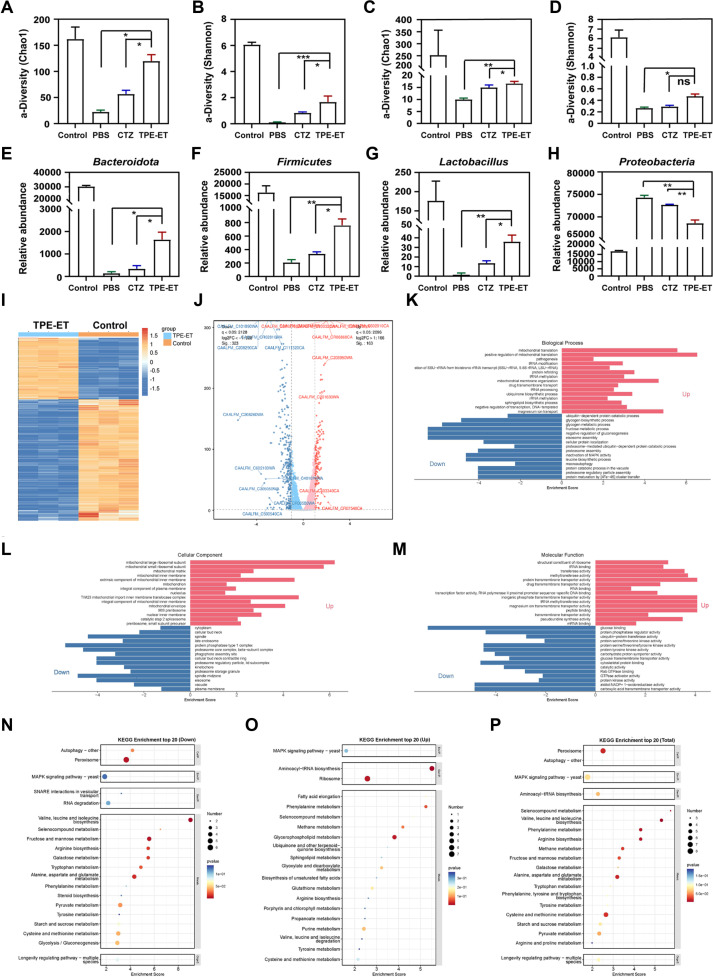
Modulation of vaginal microbial homeostasis by TPE-ET *in vivo* and transcriptomic profiles via RNA-Seq. (**A to D**) α-Diversity Sobs and Shannon indexes of the vaginal microbiome of bacteria (**A and B**) or fungi (**C and D**) in different groups in vivo (n = 3). (**E and H**) Relative abundance of *Bacteroidota* (**E**), *Firmicutes* (**F**), *Lactobacillus* (**G**) and *Proteobacteria* **H**) in different groups *in vivo* (n = 3). (**I**) Heat map of differential expressed genes in leading-edge subset expressed in TPE-ET versus control group analyzed using RNA-seq datasets (n = 3). (**J**) Volcano plot of differential expressed genes between the TPE-ET and the control group (n = 3). (**K to M**) Biological process, cell component and molecular function of top 20 GO function was applied to identify the enrichment of differential expressed genes (n = 3). (**N to P**) Bubble diagram of KEGG enriched top 20 pathways of downregulated genes (**N**), upregulated genes (**O**) and total genes (**P**). Statistical analyses were performed using one-way ANOVA (**A-H**). Error bars indicated the mean ± SD. CTZ: clotrimazole. ns: no significance, *p < 0.05, **p < 0.01, ***p < 0.001.

The compositional profile of vaginal microbiota critically governs vaginal mucosal homeostasis. Patients with *Candida* vaginitis are related to vaginal microbial dysbiosis, characterized by a decreased diversity of microbial communities, significant reduced in *Firmicutes* abundance concomitant with *Proteobacteria* expansion [32, 33]. The second ribosomal internal transcribed spacer amplicon sequencing (ITS) and 16S rRNA gene sequencing have been a mainstay in the identification of human microbial communities [34, 35]. Subsequently, vaginal washes samples in CTL, model, CTZ and TPE-ET treated groups were analyzed using ITS and 16S rRNA sequencing to compare the differences in microbial diversity between different groups. The Shannon index quantifies diversity through species richness and abundance distribution evenness. The results showed that compared with CTZ, TPE-ET treatment relatively reduced the destruction of the vaginal microbiome, recovered bacterial (Fig. 5A and 5B) and fungal (Fig. 5C and 5D) diversities in mice with *Candida* vaginitis. TPE-ET treatment markedly decreased the relative abundance of *Proteobacteria* while concurrently elevating levels of *Firmicutes* and *Bacteroidota*. At genus level, TPE-ET administration significantly enhanced *Lactobacillus* predominance and promoted vaginal microbiota restoration (Fig. 5E-5H). TPE-ET showed potential utility in the therapy and prophylaxis of vaginal dysbiosis in rodent models. Given the differences between the vaginal environment of animals and humans, the precise association between TPE-ET and human diseases warrants further investigation.

### Antifungal Mechanism

To investigate the antifungal mechanism of TPE-ET, we performed transcriptomics sequencing. As shown in Fig. 5I-5J, the results revealed 486 differentially expressed genes (DEGs) in TPE-ET treated groups compared with the CTL groups. Transcriptomic profiling identified 163 upregulated and 323 downregulated genes. We analyzed the genes with significant differential expressions, among which GPD2 showed the most pronounced fold change (Table S2). GPD2 is localized to the mitochondrial inner membrane and functions as the core enzymatic component of the glycerol-3-phosphate shuttle. By catalyzing the the oxidation reaction that glycerol-3-phosphate (G3P) to dihydroxyacetone phosphate (DHAP), it feeds electrons into the respiratory chain through the reduction of FAD? to FADH2. Consequently, GPD2-mediated metabolism is integral to cellular respiration and energy production, exerting a direct impact on mitochondrial function. DEGs with core biological processes, were including mitochondrial translation, integral component of plasma membrane and other biological processes (Fig. 5K-5M). KEGG analysis identified significant enrichment of the DEGs in mitochondrial function, metabolic processes, and inflammatory pathways (Fig. 5N-5P).

**Fig. 6:**
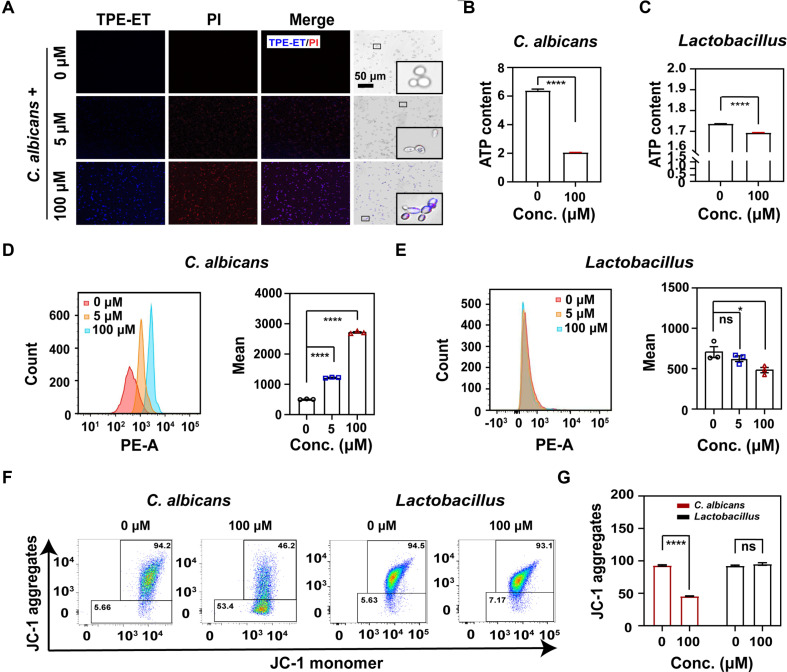
TPE-ET eliminated *C. albicans* via triggering mitochondrial dysfunction. (**A**) Confocal fluorescence images of *C. albicans* incubated with TPE-ET for 30 min and then co-stained by PI. TPE-ET possessed prominent antifungal activity against *C. albicans*. Scale bar: 50 μm. (**B and C**) Reduced ATP content of *C. albicans* and *Lactobacillus* incubated with TPE-ET (n = 3). (**D and E**) The ROS levels differed between *C. albicans* and *Lactobacillus*, with a marked increase in *C. albicans* upon TPE-ET at different concentrations (n = 3). (**F and G**) TPE-ET reduced the mitochondrial membrane potential level of *C. albicans* by flow cytometry, whereas that of *Lactobacillus* remained unaltered (n = 3). Statistical analyses were performed using two-way ANOVA (**G**), one-way ANOVA (**D, E**) and Student's t-test (**B, C**). Conc.: concentration. Error bars indicated the mean ± SD. ns: no significance, *p < 0.05, ****p < 0.0001.

With the stable aggregates, TPE-ET exhibited potent membrane-permeating properties to fast crossing the fungal cell walls. TPE-ET enabled immediate fluorescence labeling of *C. albicans*, with signal intensity scaling concentration-dependently while maintaining nearly 100% labeling efficiency. To further monitor the progress of TPE-ET destruction on the membrane surface of *C. albicans*, fluorescence imaging was performed after incubation of *C. albicans* with TPE-ET aggregates at different concentrations and co-staining with PI, which acted as a marker for the fungal membrane permeability, showed a strong destructive effect on fungal membranes (Fig. 6A). Upon disruption of the fungal biofilms, TPE-ET penetrated into fungi. RNA-seq profiling uncovered significant gene expression alterations were primarily enriched in mitochondrial function-related processes. We therefore hypothesized that TPE-ET likely targeted the fungal gene GPD2 in the mitochondrial inner membrane, thereby disrupting the metabolic-inflammatory pathway and inducing mitochondrial dysfunction, resulting in potent antifungal efficacy. Fungal mitochondria represent a potential target for fungicide development, as these organelles are involved in a range of cellular processes, including ATP production through oxidative phosphorylation[36]. Cardinal features of mitochondrial dysfunction comprised depleted organelle mass, diminished ATP output, and collapse of the mitochondrial membrane potential (MMP) [37, 38]. Therefore, we assessed changes in ATP levels, intracellular ROS, and MMP in *C. albicans* and *Lactobacillus* following TPE-ET treatment. We found that TPE-ET treatment significantly decreased the ATP content in *C. albicans*. Interestingly, we observed a decrease in ATP levels in *Lactobacillus* as well. This prompted us to investigate this unexpected phenomenon (Fig. 6B and 6C). Intracellular ROS accumulation in *C. albicans* was significantly augmented, as quantified by flow cytometry. In contrast, the ROS level in *Lactobacillus* decreased significantly, which did not lead to bacterial death but instead promoted its growth (Fig. 6D and 6E). Furthermore, measurements of membrane potential changes using the JC-1 dye after TPE-ET treatment showed that it significantly reduced the mitochondrial membrane potential in *C. albicans*, whereas the membrane potential in *Lactobacillus* remained unchanged (Fig. 6F and 6G).

## Discussion

The vagina hosts a complex microbial community, with its homeostasis serving as a natural barrier against pathogen invasion and a key factor in reproductive health. From a pathological perspective, the vaginal microbiota is essential for preserving the health and balance of the vaginal microenvironment[39]. As the predominant beneficial genus, *Lactobacillus* helps stabilize the microbial community and defends the vagina against pathogens through multiple mechanisms, including the production of lactic acid and antimicrobial compounds, the formation of a biological barrier, and the protection of vaginal epithelial cells [40, 41]. Nowadays, the pathogenesis of VVC is thought to reflect a microbiome imbalance or dysbiosis in the vagina, mainly showing a reduced proportion of Lactobacillus, accompanied by overgrowth of pathogenic *Candida* and other microorganisms [42]. As the understanding of vaginal microecology deepens, the therapeutic approach to vaginal infectious diseases has gradually shifted from a single bactericidal model in the past to a comprehensive strategy of anti-pathogen and restoration of microecological balance. This concept emphasizes that after inhibiting or eliminating pathogenic bacteria, further steps should be taken to repair the vaginal mucosa and rebuild a lactobacillus-dominated microecological environment, thereby achieving complete cure of the disease.

Existing antifungal drugs, at concentrations effective against planktonic *C. albicans*, are largely ineffective against *C. albicans* cells in biofilms [43]. Bioﬁlms, one of the main causes of drug resistance, which are consisting of fungal cells and extracellular matrix that are embedded in self-produced [44]. By providing a supportive environment for fungal proliferation, the extracellular matrix allows fungi to survive adverse conditions and evade host immunity, leading to robust resistance against antifungal drugs [45]. *C. albicans* grows and forms biofilms to adapt to a variety of microenvironment, accounting for 70% to 90% of *C. albicans* patients. As far as we know, *C. albicans* biofilms are intrinsically resistant to most known antifungal drugs [46-48]. Nowadays, therapeutic approaches that target *C. albicans* while simultaneously modulate the vaginal microenvironment and reduce drug resistance remain limited.

Unlike conventional fluorophores that suffer from aggregation-caused quenching (ACQ) due to the aggregation effect, AIEgens exhibit weak or no emission when molecularly dissolved in solution, but upon aggregation, they emit bright fluorescence. This is attributed to the synergistic effect of restricted intramolecular rotation (RIR) and a highly twisted molecular conformation, the latter of which hinders intermolecular π–π stacking interactions. For some AIEgens with luminogens containing moieties that undergo vigorous intramolecular vibrations, their aggregation-induced emission is explained by the restricted intramolecular vibration (RIV) mechanism. Intramolecular vibrations dissipate energy, whereas steric hindrance upon aggregation restricts such vibrations, thereby blocking non-radiative decay channels and increasing the fluorescence quantum yield. As the number of normal vibrational modes decreases, the loss of excited-state energy is also reduced. This unique property allows AIEgens to operate in a “turn-on” or “lighting-up” mode. Cationic amphiphiles represent a classic class of broad-spectrum agents against both bacteria and fungi. Their mechanism targets non-genetic sites, which is thought to make them less prone to inducing genetic mutations or promoting drug resistance [49]. Another claimed advantage is their high selectivity for microbial cells over mammalian cells [50, 51]. However, the non-fluorescent nature of amphiphile aggregates hinders the acquisition of fluorescence-based information on their interaction with fungi. Unlike conventional fluorophores that suffer from aggregation-caused quenching (ACQ), aggregation-induced emission luminogens (AIEgens) are weakly or non-emissive in solution but become strongly fluorescent upon aggregation [52]. This unique property enables AIEgens to function in a light-up or turn-on mode [53]. TPE is a notable AIEgen that is widely studied for its rich functional group modifiability, excellent thermal stability, reliable biocompatibility and facile chemical modification [54]. To systemically engineer cationic amphiphile hydrophilicity and aggregate stabilities, structurally, they feature a positively charged ammonium group paired with tailored hydrophobic moieties, allowing precise modulation of their electrostatic and hydrophobic interactions with pathogens. To achieve this, we developed an AIE-active TPE derivatives, designated as TPE-ET.

In this study, TPE-ET could preferentially and selectively light up *C. albicans* rather than *Lactobacillus* and normal cells. TPE-ET inhibited the formation of biofilms to reduce drug resistance by downregulating the expression of genes associated with the adhesion, invasion and drug resistance of *C. albicans*. Additionally, after vaginal perfusion of TPE-ET, the dark antifungal activity endowed it with an excellent killing efficiency to *C. albicans* in mice models. TPE-ET displayed an excellent anti-infection capability against *C. albicans*. Compared with the clinically used CTZ, TPE-ET significantly alleviated inflammation, with nearly complete remission of erythema, edema, and pathological discharge. Mucosal defects and inflammation of the vaginal mucosal tissue were markedly improved. We employed ITS and 16S sequencing to investigate whether TPE-ET could modulate the vaginal microenvironment and restore a healthier vaginal microbiota. The results indicated that TPE-ET was superior to clotrimazole in re-establishing a healthy microecology, as evidenced by a reduced abundance of *Proteobacteria* and increased abundances of *Firmicutes* (particularly *Lactobacillus*) and *Bacteroidetes*.

To gain deeper insights into its mechanism of action, we performed comparative transcriptomic analysis of *C. albicans* to elucidate the differential gene expression between TPE-ET treated and untreated groups, thereby further uncovering its antimicrobial mechanism. Subsequent functional enrichment analyses revealed significant associations of these DEGs with core biological processes, including mitochondrial translation, integral component of plasma membrane and other biological processes. According to KEGG pathway analysis, the differentially expressed genes exhibited significant enrichment in mitochondrial function, metabolic processes, and inflammatory pathways. We investigated the changes in intracellular ATP levels, ROS levels, and membrane potential of *C. albicans* and *Lactobacillus* following TPE-ET treatment. We found that ATP levels decreased in both *C. albicans* and *Lactobacillus*. However, intracellular ROS accumulation significantly increased in *C. albicans*, whereas ROS levels decreased in *Lactobacillus*. Notably, *Lactobacillus* did not undergo cell death due to TPE-ET intervention; instead, its growth was promoted. The mitochondrial membrane potential of *C. albicans* was significantly reduced, while that of *Lactobacillus* remained unchanged. We suspected that it might be TPE-ET exposure induced environmental alterations, leading to transient inhibition of metabolic enzyme activity which caused temporary ATP depletion in *Lactobacillus*. Crucially, our preceding PI staining confirmed intact cell membranes post-treatment. Furthermore, both plate assays and ROS detection indicated no significant viability impairment. Collectively, these findings suggest the observed ATP reduction represents a reversible response to environmental perturbation rather than cellular damage. Taken together, these results indicate that TPE-ET induces mitochondrial dysfunction in *C. albicans*.

In light of the strong therapeutic efficacy of TPE-ET against VVC, a new antifungal approach leveraging its dark antifungal activity is expected to provide high efficiency against VVC. Such a strategy could help mitigate the widespread drug resistance associated with conventional antifungal treatments in clinical settings, and may also be extended to other fungal infections.

## Supplemental Materials

Supplementary data for this paper are available on-line only at http://jmb.or.kr.
