# Supplementary material for: Charge-Competition AIEgens Induce Mitochondrial Dysfunction for Selective Eradication of Candida albicans while Restoring Vaginal Microbiota
Source: J Microbiol Biotechnol. 2026 Jun 12;36:e2601074. doi: 10.4014/jmb.2601.01074 (PMC13265238; doi:10.4014/jmb.2601.01074)
Supplement: Supplementary file 1 [file jmb-36-e2601074-supple.pdf]

J. Microbiol. Biotechnol. 2026. 36: e2601074

https://doi.org/10.4014/jmb.2601.01074

Supplementary Figures and Tables

Charge-Competition AIEgens Induce Mitochondrial Dysfunction for Selective Eradication of *Candida albicans* while Restoring Vaginal Microbiota

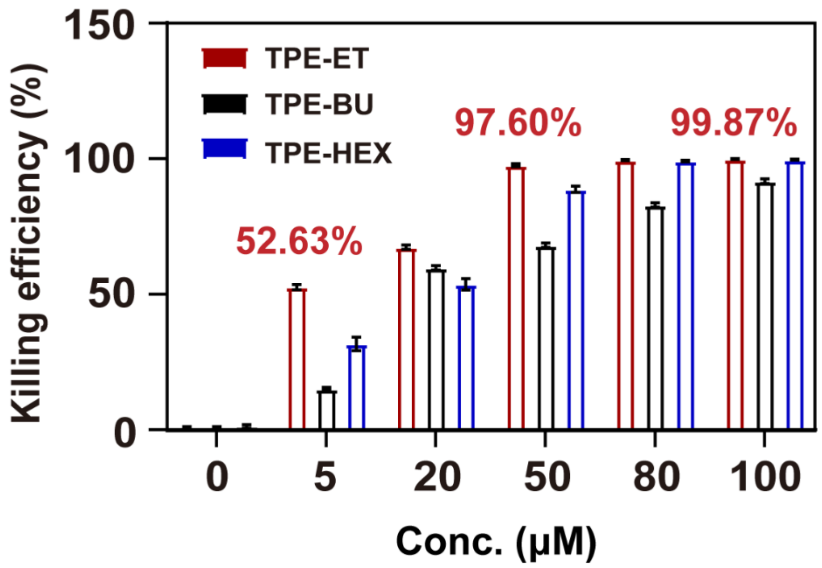

Fig. S1. **The killing *C. albicans* efficiency of TPE-BU, TPE-HEX, TPE-ET with different concentrations. Conc.: concentration.**

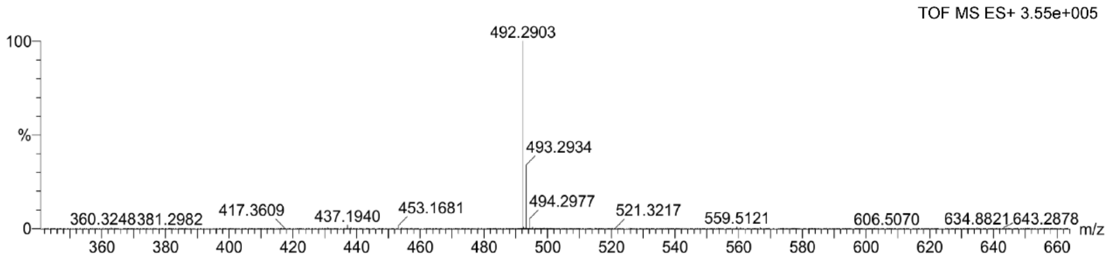

Fig. S2. **HRMS spectra of TPE-ET.**

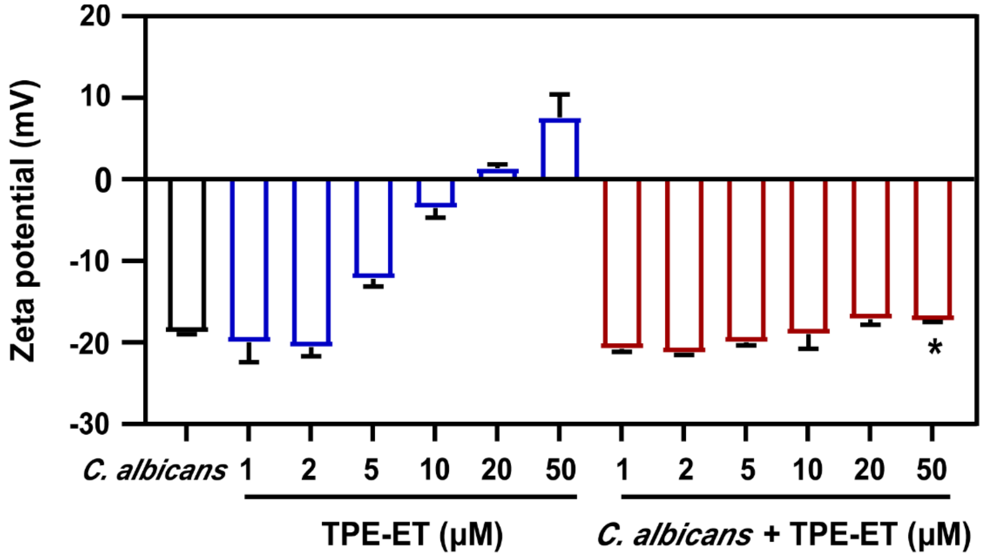

Fig. S3. **Zeta potential results of *C. albicans* before and after incubated by different concentrations TPE-ET for 30 min (n=3).** *C. albicans* exhibited a reduced absolute zeta potential in comparison to the untreated control upon 50 μM TPE-ET intervention. Statistical analyses were performed by Student's t-tests. *p<0.05.

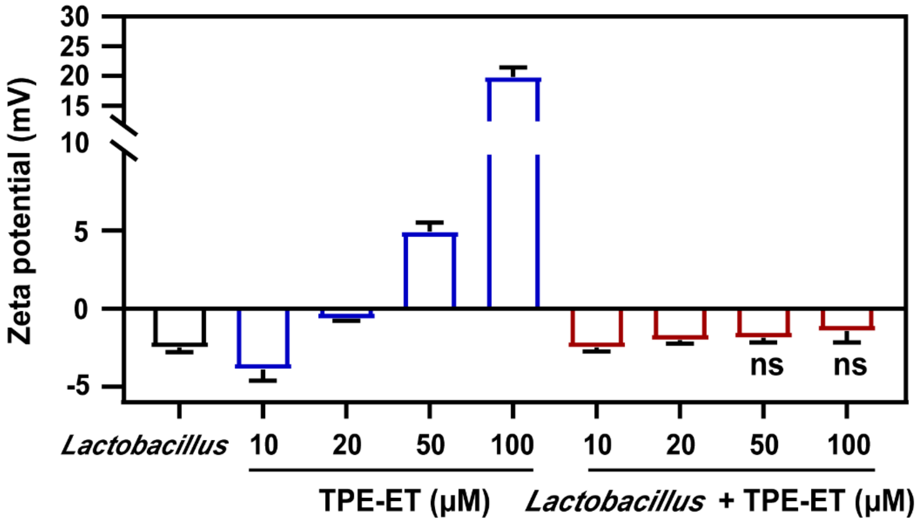

Fig. S4. Zeta potential results of *Lactobacillus* before and after incubated by different concentrations of TPE-ET for 30 min (n=3). TPE-ET (50 μM or 100 μM) failed to alter the zeta potential of *Lactobacillus.* Statistical analyses were performed by Student's t-tests. ns: no significance.

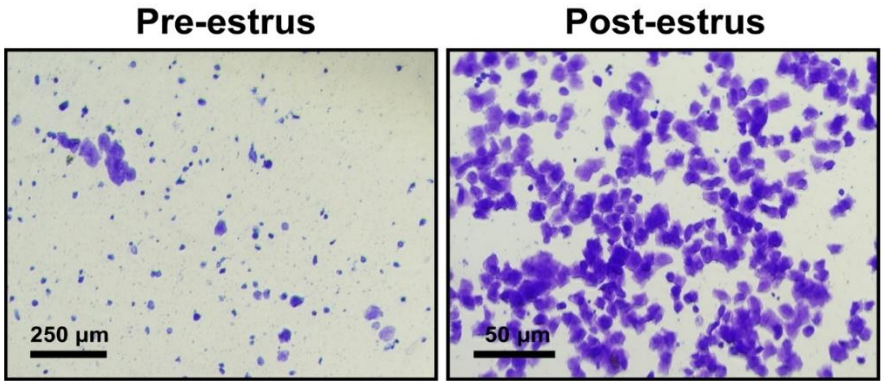

Fig. S5. Vaginal smears of mice during pre-estrus and post-estrus phases. Scale bar: 250 μm (left), 50 μm (right).

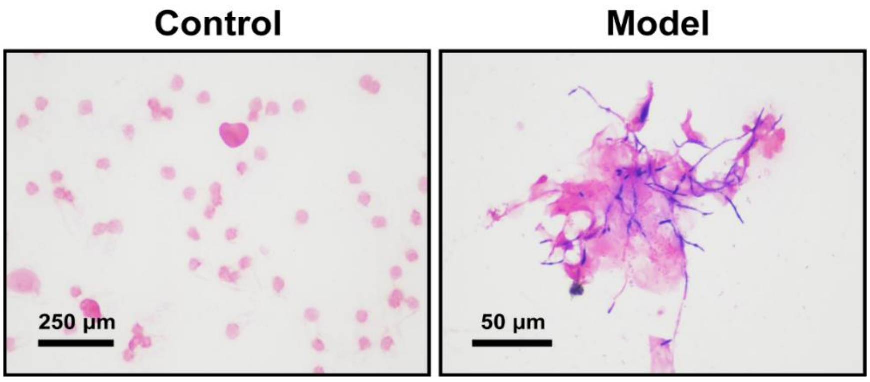

Fig. S6. **Gram stain smear of vaginal lavage fluid from mice after injecting *C. albicans.*** Scale bar: 250 μm (left), 50 μm (right).

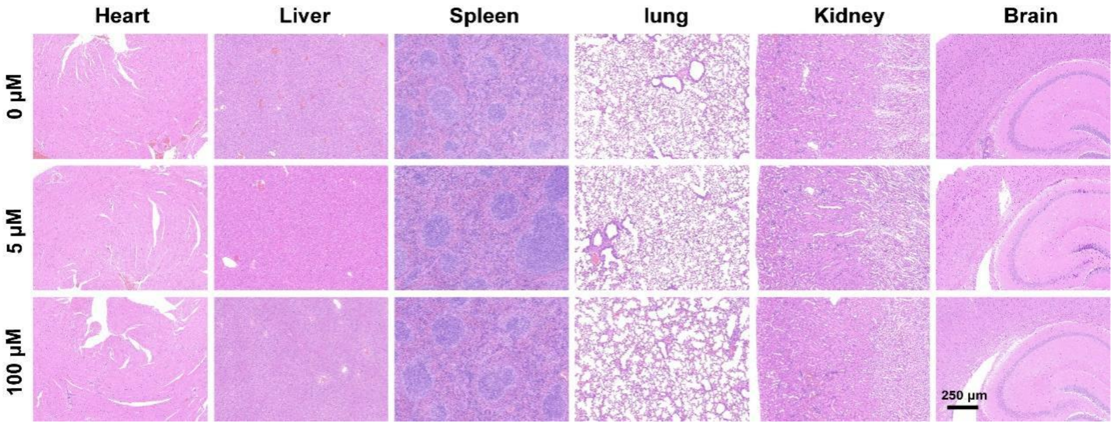

Fig. S7. **HE staining of the heart, liver, spleen, lung, kidney and brain of mice after TPE-ET treatment with different concentrations**. TPE-ET exhibited no significant toxic effect on the aforementioned vital organs. Scale bar: 250 μm.

Table S1. Primer sequences used in qRT–PCR.

qRT-PCR primers	Fp (5' to 3')	Rp (5' to 3')
IL-4	GATAAGCTGCACCATGAATGAGT	CCATTTGCATGATGCTCTTTAGG
IL-6	CCCCAATTTCCAATGCTCTCC	CGCACTAGGTTTGCCGAGTA
IL-1β	GCTTCAGGCAGGCAGTATCA	AATGGGAACGTCACACACCA
SAP1	GAACCAAGGAGTTATTGCCAAGA	TTTGTCCAGTGGCAGCATTG
SAP2	GTCACTTTAAAAAAACAAGGAGTC ATTG	TATTTGTCCCGTGGCAGCAT
ALS3	CTGGACCACCAGGAAACACT	ACCTGGAGGAGCAGTGAAAG
EAP1	TGTGATGGCGGTTCTTGTTC	GGTAGTGACGGTGATGATAGTGA CA
HWP1	CGGAATCTAGTGCTGTCGTCTCT	CGACACTTGAGTAATTGGCAGAT G
MDR1	AGTTGCTTGGGGTAGTTCCG	TGCTCTCAACTTTGGTCCGT
GAPDH	CCTCGTCCCGTAGACAAAATG	TGAGGTCAATGAAGGGGTCGT

Table S2. Significant genes alteration of *C. albicans* following TPE-ET versus untreated control.

Gene (Down)	Fold Change	log_2Fold Change	p-value	q-value	Gene (Up)	Fold Change	log_2Fold Change	p-value	q-value
GPD2	6.14930	2.62042	0.0000	0.0000	***HSP30***	0.06753	-3.88841	0.0000	0.0000
AAH1	4.99867	2.32154	0.0000	0.0000	***UCF1***	0.06933	-3.85037	0.0000	0.0000
ARO10	4.03408	2.01224	0.0000	0.0000	***TRY4***	0.09879	-3.33939	0.0000	0.0000
RHR2	4.02632	2.00945	0.0000	0.0000	***HGT17***	0.10710	-3.22296	0.0000	0.0000
GPD1	3.96539	1.98746	0.0000	0.0000	***CIRT4B***	0.12295	-3.02386	0.0000	0.0000
ROD1	3.77616	1.91692	0.0000	0.0000	***MDM34***	0.13804	-2.85689	0.0000	0.0000
